# Two-echelon inventory location model with response time requirement and lateral transshipment

**DOI:** 10.1016/j.heliyon.2022.e10353

**Published:** 2022-08-20

**Authors:** Unanaowo Nyong Bassey, Samuel Chiabom Zelibe

**Affiliations:** a Department of Mathematics, University of Ibadan, Ibadan, Nigeria; b Department of Mathematics, Federal University of Petroleum Resources, Effurun, Nigeria

**Keywords:** Inventory-location, Response time, Inventory control, Base-stock policy, Supply chain

## Abstract

This study presents a new model for a two-echelon location-inventory system with response time constraints. This system controls inventory with a (S-1, S) policy and comprises of a finite collection of customers, a finite collection of service facilities and a single plant. This paper's main novelty is the incorporation of lateral transshipment into a two-echelon location-inventory system with response time requirement. By using a continuous-time Markov process approach, we determine expected on-hand inventory level in steady state, expected lateral transshipment level in steady state and expected backorder level in steady state. We utilize these steady state levels to formulate a mixed integer nonlinear programming model which incorporates lateral transshipment into an integrated location-inventory system with response time constraint. The model minimizes the total system cost and simultaneously determines: optimal location and number of service facilities, the optimal assignment of customers and base-stock level. We exploit the model's properties using Lagrange decomposition and we show that the model is convex. The model is tested on a real-world scenario using GAMS and our model returned lower costs following comparisons with a model without lateral transshipment. We also establish that lateral transshipment results to consistency of expected cost with varying response time requirement.

## 1 Introduction

Industries which deal with the manufacture of heavy machinery, technologically advanced equipment and capital intensive items such as aircrafts, trucks, and electronics, etc, are often faced with stochastic demand from customers in need of urgent replacement or repair of failed critical parts. Users of these equipment will almost surely experience the malfunction of certain critical parts at random times and are always have very high response time sensitivity to malfunction of critical parts. The sensitivity to service times of customers in need of replacement parts is mainly driven by the desire to get their machines back to full functionality in the shortest possible time. This highlights the need for manufacturers to provide fast and efficient after-sales services which ensure availability of service parts to customers who are spread geographically. Thus, decision makers are always looking out for more efficient after-sales supply systems that factor in response time requirements of the customers and the budget constraints of the service parts resupply system. This implies that the design of after-sales supply systems which are more efficient than existing ones remains an interesting problem in research and in the real world.

Optimisation of after-sales services in service parts supply chains has been considered from various angles. One of such angles is the use of multi-echelon systems for inventory control. The paper by [1] considered a mathematical model applicable to the control of recoverable or repairable items called “Multi-Echelon Technique for Recoverable Item Control (METRIC).” The METRIC model is very influential and widely applicable in multi-echelon inventory studies because it uses an approximation to the distribution of items in replenishment to circumvent the computationally burdensome exact representation. The METRIC model is used to get an approximate expected backorder value for each facility. [2] considered a model which determines inventory stock level in multi-echelon systems. He presented an exact method for deriving expected inventory level which gave better accuracy than METRIC under same problem structure. The obvious disadvantage of his approach is that it is computationally onerous. For full treatment of inventory in multi-echelon arena including see [3]. A very important factor which affects after-sales services is response time; shorter response times are more appealing to customers. The time it takes to replace failed components is a key service requirement from customers. Motivated by this requirement, a number of studies considered multi-echelon service parts systems with time-dependent service levels. Most papers that considered service measures used the fill rates as service measure, for example [4], [5], and [6]. While a few have considered a response time threshold as the service constraint, for example [7], [8], and [9]. [8] studied a two-echelon inventory model for expensive spare parts using continuous (S-1,S) policies at both echelons. They imposed a requirement on expected time between the arrival and fulfillment of demand and created efficient algorithms to determine optimal stock level for both echelons. [10] recently presented a review of 148 research works spanning 11 years (2010-2020). They analyzed various spare parts inventory systems and classified existing literature based on analytic procedure. They also discussed existing research gaps in this field. [11] considered a two echelon inventory system with service consideration and lateral transshipment. Their model focused on just inventory decisions while the model presented in this study considers systems where decisions on facility location and inventory are integrated. Thus, the integration of location and inventory decisions makes our model different from the model presented by [11].

Integrating inventory and location decisions in systems designed for service parts logistics is another strategy which has been considered for the optimisation of after-sales services. It used to be the convention to consider decisions on inventory and location separately. One of the first studies to integrate consideration of inventory decisions and location decisions is [12]. They incorporated implicit consideration of inventory levels into the location problem without capacity constraint. [13] obtained an approximation which merged costs related to inventory with facility costs. They also proposed a model which maximized service coverage. This was extended by [14] to consider minimisation of costs under the constraints of service coverage. [15] considered locating distribution centers in a two echelon environment that holds inventory at a plant and at various distribution centers. [16] presented a nonlinear integer program which explicitly integrates inventory costs into a facility location model, this is most likely the first paper to do so. They utilised Lagrangian relaxation to solve their model. [17] solved a similar problem using a method based on column generation. [18] extended earlier studies by considering tradeoffs between costs and services in inventory-location problems. [19] showed that simultaneous consideration of location and inventory decisions yield lower system cost compared to when both decisions are considered separately. Thus, considering facility location decisions independent of decisions on inventory could result in supply chain designs that are suboptimal. Research on joint location-inventory systems further evolved to consider integrated location-inventory problems in two-echelon systems with service time constraints [9], [20]. [9] studied a two-echelon problem on integrated inventory-location decisions with service consideration. They modelled the manufacturing process as a queue and formulated a nonlinear mixed integer problem. They solved the problem using a Lagrange heuristic. [21] considered a location-inventory model with customer-based service constraints.

Another strategy of interest which has been considered for optimising after-sales services is Lateral Transshipment (LT), this is the sharing of stock between neighbouring facilities on same echelon. [22] considered a semi-conductor firm ASML under a reactive LT setting, and shows that incorporating Lateral Transshipments (LTs) resulted to annual savings of up to 50% savings of total service parts inventory cost. The use of LT has been considered in diverse multi-echelon environments in supply chain research. [23], [24], [25], [26], and [4] considered the use of LT in two-echelon inventory problems with continuous (S-1,S) replenishment policy. [6] considered time-dependent service levels, they examined cost and service level within two and three location networks. Their evaluation model highlighted the advantages of time-based constraints over fill rates, they also showed that the performance of response time improved with the use of emergency lateral transshipment. [4] considered a service parts inventory location problem having lateral transshipment and flexible replenishment stock. They proposed a customer friendly service measure and provided an approximation for optimal inventory allocation, subject to this measure. However, their customer oriented service measure did not take all possible demand into consideration, that is, there exists a possibility that a few customers will be served after the set service time window. [27] gave a comprehensive review on LT literature. [23], [24], [28], [29], [30] and [31] assume negligible LT times which happens to be a major assumption in this study.

The problem considered in this paper is a hybrid of two-echelon inventory location problems and LT. From what we know, in the literature of service parts supply systems, the incorporation of LT into a two-echelon inventory-location system with response time constraints has not been studied; this is the gap this study intends to fill. Hence, this study was designed to incorporate lateral transshipment into a two-echelon system which jointly minimises expected costs emanating from facility location decisions and inventory decisions constrained by a requirement on response time.

In this study, we present a mathematical model that simultaneously determines optimal number of Service Centres (SVCs), optimal location of SVCs, optimal customer assignments to SVCs and optimal inventory levels (on-hand inventory level, LT level and backorder level); constrained by a requirement on response time. We also examine some mathematical properties of the model and use GAMS 32.1.0 to perform some computation experiments that show the effects of LT. Our two-echelon LT system consists of a plant for manufacturing with production and capacity constraints and a collection of SVCs which satisfy demand from geographically dispersed customers. The SVCs replenish inventory from the plant. In this study, LT only takes place within SVCs in same pool; SVCs which are within defined neighbourhoods are pooled together to form a pool. For service parts supply chains, multi-echelon environments are ideal for geographically dispersed customers. This is because the sensitivity of customers to time means that customers who are far from the manufacturing plant might not be tolerant to the possibility of longer delays if their orders have to be shipped directly from the manufacturing plant. Our two-echelon system uses a base-stock policy for both echelons. Base-stock (S -1, S) policies are apparently fitting for slow moving products that have high holding costs. [32] analytically check base-stock policy optimality given specific problem parameters. Their findings imply that the optimality of base-stock policy holds in a setting that admits low rate of demand and low setup costs in comparison with holding costs. The customer service considered in this study is a requirement on response time; where the service provider reaches an agreement with customers on favourable response time thresholds.

The major contributions made by this study are enumerated below:

1. We partitioned all lower echelon facilities into pools and establish steady state properties of a two-echelon inventory-location system with lateral transshipment and a requirement on response time; lateral transshipment can only take place between facilities in same pool.
2. We used the steady state properties to formulate a new model which incorporates lateral transshipment into a two-echelon location-inventory centralised system with finite number of lower echelon facilities and a requirement on response time across all facilities.
3. We showed that this new model is convex and also carried out some computational experiments in order to highlight other features of the model and draw managerial insights.

From what we know, this is the first paper that incorporates LT into the model of [9]. The incorporation of lateral transshipment causes some change in dynamics with respect to methodology. The introduction of pooling resulted to significant differences in expressions used for our steady state properties and other model properties. For, example, the response time constraint is pool dependent and is clearly different from that of [9].

The paper is structured in the following way. In Section 1, we present the introduction. We present the problem description and formulation of the joint model in Section 2. In Section 3, we derive the optimal inventory, backorder and LT levels. In Section 4, we examine some analytic properties of the model. In Section 5, we present computational experiments to highlight the effects of LT. In Section 6 we present our conclusion.

## 2 Problem description and model formulation

We begin this section with a description of the problem and proceed to present notations used in this study. We end this section by presenting a basic formulation of our model.

### 2.1 Problem description

**Figure 1:**
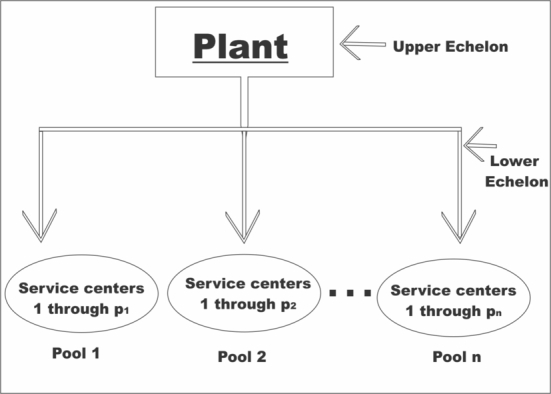
Two-echelon problem structure.

In this subsection, we describe a real-world problem which could arise in a two-echelon supply chain with simultaneous consideration of inventory and location decisions. Our two-echelon system deals with a single item, the top echelon is made up of a plant, while the lower echelon comprises of a collection of SVCs and a collection of customers. The problem considered in this paper is domiciled in the arena of spare parts logistics. We begin by presenting a general description of the problem. This is followed by a presentation of notations and key assumptions. We end this section by presenting a basic formulation of a model which incorporates lateral transshipment into a two-echelon inventory-location model with service constraints. Fig. 1 describes the two-echelon problem structure considered in this study.

We proceed to give a detailed description of the problem.

1. The item is manufactured at the plant and the manufactured items are held at the plant to satisfy Service Centre (SVC) demands. The plant resupplies the SVCs within a SVC specific replenishment lead time.
2. The SVCs keep inventory to satisfy orders from customers. Each customer is assigned to one and only one SVC by a central decision maker and customer orders placed at an assigned SVC follow a Poisson process. Orders by different customers are independent, hence demand processes at various SVCs are independent Poisson processes.
3. The plant and all SVCs have limited storage spaces for holding inventory and are controlled using continuous (S−1,S) replenishment policies.
4. When a customer order arrives, the SVC satisfies this order from its on-hand inventory (if inventory position is positive) and places an order for replenishment with the plant immediately.
5. If the SVC has zero or negative stock level at the SVC, the customer will wait for the part and the request will be satisfied by LT from pooled neighbouring SVCs which have stock on hand.
6. If none of the pooled neighbouring SVCs have stock on hand, demand is backordered.
7. We assume that LT is instantaneous. [23], [24], [33], and [34] also considered instantaneous LT in previous works.
8. The SVC that serves a customer is not determined by the customer's preference. This and the assumption of instantaneous LT imply that a customer cannot choose to go to another SVC in the event of a stockout at her assigned SVC. Instead, in the event of a stockout at a customer's assigned SVC, the customer's demand is then assigned to another SVC in same pool.
9. When an order for replenishment from a SVC arrives the plant, the plant satisfies that order from its available stock, and instantly triggers a production order for one unit. The plant possesses a single production line.
10. If the plant's inventory level is zero, demand arrival is backordered.
11. Items produced at the plant's production line are either used to offset existing backorders or they are kept in storage at the plant. It is assumed that the plant has an exponential service rate. Since each SVC faces a superposition of Poisson demand from customers and places replenishment orders in a one-to-one manner, the plant faces Poisson demand. This then implies that the production line has the characteristics of a Markovian queue.
12. All demands at the SVCs, replenishment orders at the plant, transshipment and backorder requests are treated in a First-Come, First-Served (FCFS) manner.
13. Demand arrivals at the SVC are fulfilled from stock on hand, lateral transshipment or backorder.

Our model considers the following costs:

1. fixed SVC location costs (cost incurred as a result of opening SVCs),
2. costs emanating from holding inventory at opened SVCs and at the plant,
3. backorder costs at SVCs (costs incurred when demand at a SVC is backordered), and
4. lateral transshipment costs at SVCs (cost incurred when demand is met via lateral transshipment).

### 2.2 Key assumptions

Two key assumptions are made concerning the model. They are:

1. Lateral transshipment times are negligible. [23], [24], [28], [29], [30] and [31] are some previous works with the assumption of negligible transshipment times. The consequence of the assumption on our model is that the deterministic transportation time from the central plant is identical for all SVCs in a pool; this is also another notable difference between our model and that of [9].
2. The SVC basestock level is identical for all SVCs in a pool. A common feature of many lateral transshipment models is that all parameters are identical for facilities in same pool. Our model is different because demand rate is not identical.

The problem considered in this work is a hybrid of inventory and facility location problems. We now present the joint model.

### 2.3 Joint location and two-echelon inventory model with lateral transshipment and service consideration

Here, we show the full formulation of our model.

(1) $$\begin{array}{c} \min\sum_{w\in W}\sum_{v\in V}\left(f_{vw}X_{vw}+h_{vw}I_{vw}+p_{vw}B_{vw}+q_{vw}T_{vw}+\sum_{u\in U}\lambda_{u}Y_{uvw}d_{uvw}\right)+h_{0}I_{0} \end{array}$$

Subject to:

(2) $$\sum_{v\in V}Y_{uvw}=1,\;\text{for any,}\;u\in U$$

(3) $$Y_{uvw}\leq a_{uvw}X_{vw},\;\text{for any,}\;u\in U,v\in V$$

(4) $$S_{vw}\leq C_{vw},\;\text{for any,}\;v\in V$$

(5) $$S_{w}\leq |w|C_{vw},\;\text{for any,}\;w\in W$$

(6) $$S_{0}\leq C_{0}$$

(7) $$Wt_{vw}\leq \tau ,\;\text{for any,}\;v\in V$$

(8) $$S_{vw}\geq 0,integer,\;\text{for any,}\;v\in V$$

(9) $$S_{0}\geq 0$$

(10) $$X_{vw}\in \{0,1\}\;\text{for any,}\;v\in V$$

(11) $$Y_{uvw}\in \{0,1\}\;\text{for any,}\;u\in U$$

The objective function (1) is to find the minimum sum of the fixed location costs, holding costs for plant inventory, holding costs for inventory at the SVCs, backorder costs at SVCs, lateral transshipment costs at SVCs and transportation costs. Constraints (2) state that all demand should be assigned to SVCs. Constraints (3) require that a candidate location cannot be assigned demand from a customer except a SVC is sited at that location and the distance from the customer under consideration is less than $d_{max}$. Constraints (4), (5) and (6) state that base-stock at SVCs, pools and plant cannot be greater than their respective capacities. The constraints on service times (7) place a threshold on response times. Finally, (8), (9), (10), and (11) are nonnegativity constraints on base-stock levels and integer constraints on location and assignment variables.

The service requirement in this model is that expected response time across all SVCs must not be greater than the guarantee in the customer contract (7). In this two-echelon model, response time at any SVC with stock-outs may be short if any of its pooled SVCs have stock on hand and ships out immediately. In a situation in which all the pooled SVCs are also experiencing stock-outs, the response time would also be short if the plant's inventory on hand is positive and the plant ships without any delay. However, if a stock-out situation occurs at the plant, a longer response time will occur. A requirement on response time is appropriate for this study since we are dealing with the design of an inventory system for service parts.

Each SVC has the characteristics of a queuing system [35] in which the customer orders can be regarded as the items in the system. Then the number of items awaiting service in the system, represents the backorder level and the time the item spends in the system represents the service time. [35] derived the waiting time and applied Little's law to show that a SVC's expected response time is given by

$$Wt_{vw}=\frac{B_{vw}}{\sum_{u\in U}\lambda_{u}Y_{uvw}}$$

We may replace (7) with the following:

$$B_{vw}\leq \tau \sum_{u\in U}\lambda_{u}Y_{uvw}$$

## 3 Steady state inventory levels

The plant's steady state inventory level is presented in this section; steady state levels are also presented for the pools and SVCs.

### 3.1 Steady state inventory levels at the plant

Merging of independent Poisson processes implies that the arrival process at each SVC is Poisson. This follows since demand arrivals at a SVC follow a Poisson process and are independent. Operating each SVC with a (S-1,S) policy imply that a replenishment request is placed on the plant by a SVC immediately a demand arrival occurs. Hence, the plant's demand arrival process is a merging of independent Poisson processes and is thus, also a Poisson process. The plant possesses a single production line. With plant service rate being exponential, the plant exhibits the characteristics of a queuing system with demands considered as arrivals and in-replenishment items are considered to be in service. Let $N_{0}$ represent the steady state order quantity in the entire queuing system.

The plant's order arrivals are satisfied through inventory at hand or through backorders in the event of a stockout. The plant's total demand is given by $\lambda_{0}=\sum_{w\epsilon W}\lambda_{W}=\sum_{w\in W}\sum_{v\in V}\lambda_{vw}=\sum_{w\in W}\sum_{v\in V}\sum_{u\in U}\lambda_{u}Y_{uvw}$, the outstanding orders in the plant (orders still in queue and orders being attended to) $N_{0}$ with mean $E[N_{0}]$. Using Palm's theorem [36], [8] established the following standard expressions for steady state levels for inventory on-hand and backorder.

The plant's on-hand inventory level in steady state is

$${\overset{¯}{I}}_{0}=S_{0}-E[N_{0}]+{\overset{¯}{B}}_{0}.$$

The plant's backorder level in steady state is

$$B_{0}=E[N_{0}]-\sum_{s=0}^{S_{0}-1}[1-F_{0}(s)],$$

where

$$F_{0}(s)=\sum_{m=0}^{s}P\{N_{0}=m\}.$$

The plant's expected levels for inventory and backorder are easily obtainable for different manufacturing queue systems via the substitution of steady state probabilities into the formulas above. For the M/M/1 case, we state the result by [37], who showed that the steady state plant backorder level is given by

$$B_{0}=\frac{\rho^{S_{0}+1}}{1-\rho }$$

and the steady state plant on hand inventory is given by

$$I_{0}=S_{0}-\frac{\rho }{1-\rho }(1-\rho^{S_{0}})$$

From [9],

$$W_{0}=\frac{\rho^{S_{0}+1}}{\lambda_{0}(1-\rho )}$$

### 3.2 Steady state inventory levels at the pool

Demand faced by each pool is satisfied either through pool inventory at hand or through backorders. Just like the plant, the pool inventory level in steady state is

$$I_{w}=\sum_{s=0}^{|w|S_{vw}-1}(|w|S_{vw}-s)P\{N_{w}=s\}$$

or

$$I_{w}=\sum_{s=0}^{|w|S_{vw}-1}F_{w}(s)$$

where

$$F_{w}(s)=\sum_{m=0}^{s}P\{N_{w}=m\}$$

By the model assumptions, backorders can only occur in pool w if all SVCs in that pool are out of stock. If all pools are treated as single facilities, our system becomes similar to that of [9]. Then expected backorder level in steady state at each pool is

$$B_{w}=E[N_{w}]-\sum_{s=0}^{|w|S_{vw}-1}[1-F_{w}(s)]$$

$$=E[N_{w}]-|w|S_{vw}+\sum_{s=0}^{|w|S_{vw}-1}F_{w}(s)$$

The distribution in steady state of outstanding orders in pool w $N_{w}$, is required to determine the levels for backorder and inventory. At a point in time t, the number of outstanding orders from pool w comprises of:

(a) plant backorders due to pool w at time $t-\alpha_{w}$ (this quantity will not reach pool w before t because they had a backorder status at time $t-\alpha_{w}$ and as such were not shipped immediately) and

(b) the number of new orders made during the interval $\left(t-\alpha_{w},t\right)$.

Suppose the plant processes orders in a First Come First Served (FCFS) manner, the splitting of Poisson processes implies that plant backorders can be disaggregated randomly. This implies that the probability that a backorder belongs to a particular pool is proportional to the demand rate (i.e. reorder rate) faced by the pool. The expected number of the backorder due to pool w in steady state is given by $(\frac{\lambda_{w}}{\lambda_{0}})B_{0}$.

The expected number of new orders made in a time interval of length $\alpha_{w}$ is $\lambda_{w}\alpha_{w}$. Therefore expected number of $N_{w}$ in steady state is given by:

$$E[N_{w}]=\frac{\lambda_{w}}{\lambda_{0}}B_{0}+\lambda_{w}\alpha_{w}=\frac{\lambda_{w}}{\lambda_{0}}\frac{\rho^{S_{0}+1}}{1-\rho }+\lambda_{w}\alpha_{w}=\lambda_{w}L_{w}$$

In the above, $L_{w}$ is the expected lead time for replenishment. $L_{w}$ comprises of the plant's expected response time and the plant to pool w lead time.

$$L_{w}=W_{0}+\alpha_{w}=\frac{\rho^{S_{0}+1}}{\lambda (1-\rho )}+\alpha_{w}$$

Therefore,

$$B_{w}=\lambda_{w}L_{w}-|w|S_{vw}+\sum_{s=0}^{|w|S_{vw}-1}F_{w}(s)$$

Where $\lambda_{w}=\sum_{v\in V}\sum_{u\in U}\lambda_{u}Y_{uvw}$.

### 3.3 Steady state inventory level at a SVC

If a SVC's inventory level is greater than zero, demand faced at that SVC is satisfied through on-hand inventory. If the inventory level is zero, the demand arrival at the SVC is satisfied via LT from any other SVC in same pool without zero inventory level. Backorders occur if all SVCs in the pool have zero inventory level. Thus, SVC demand is satisfied from any one of inventory on hand, LT, and backorder.

We begin by establishing a result which determines the steady state inventory level, LT level and backorder level at a SVC. This result builds on work done by [8] and [37].

Proposition 3.3.1

1. *For each SVC v in pool w, the expected inventory level in steady state is*
  (12) $$I_{vw}=S_{vw}-E[N_{vw}]+T_{vw}+B_{vw}$$
2. *For each SVC v in pool w, the expected backorder level in steady state is*
  (13) $$B_{vw}=I_{vw}-S_{vw}+E[N_{vw}]+T_{vw}$$
3. *For each SVC v in pool w, the expected lateral transshipment level in steady state is*
  (14) $$T_{vw}=I_{vw}-S_{vw}+[N_{vw}]-B_{vw}$$

Proof $$I_{vw}=\sum_{s=0}^{S_{vw}-1}(S_{vw}-s)P\{N_{vw}=s\}=S_{vw}\sum_{s=0}^{S_{vw}-1}P\{N_{vw}=s\}-\sum_{s=0}^{S_{vw}-1}sP\{N_{vw}=s\}=S_{vw}(1-\sum_{s=S_{vw}}^{\infty }P\{N_{vw}=s\})-(\sum_{s=0}^{\infty }sP\{N_{vw}=s\})-\sum_{s=S_{vw}}^{\infty }sP\{N_{vw}=s\})=S_{vw}-S_{vw}\sum_{s=S_{vw}}^{\infty }P\{N_{vw}=s\}-(E[N_{vw}]-\sum_{s=S_{vw}}^{\infty }sP\{N_{vw}=s\})=S_{vw}-E[N_{vw}]+\sum_{s=S_{vw}}^{|w|S_{vw}}(s-S_{vw})P\{N_{vw}=s\}\;+\sum_{s=|w|S_{vw}+1}^{\infty }(s-|w|S_{vw})P\{N_{vw}=s\}=S_{vw}-E[N_{vw}]+T_{vw}+B_{vw}$$

This proves (12). Making $B_{vw}$ and $T_{vw}$ the subject of the formula yields (13) and (14) respectively. □

Proposition 3.3.2

1. *For each SVC v in pool w, the expected inventory level in steady state is*
  $$I_{vw}=\sum_{s=0}^{S_{vw}-1}(S_{vw}-s)P\{N_{vw}=s\}$$
  *where*
  $$F_{vw}(s)=\sum_{m=0}^{s}P\{N_{vw}=m\}$$
2. *For each SVC v in pool w, the expected backorder level in steady state is*
  $$B_{vw}=\sum_{u\in U}\lambda_{u}Y_{uvw}L_{w}+\frac{\sum_{u\in U}\lambda_{u}Y_{uvw}}{\lambda_{w}}\left(\sum_{s=0}^{|w|S_{vw}-1}F_{w}(s)-|w|S_{vw}\right)$$
3. *For each SVC v in pool w, the expected lateral transshipment level in steady state is*
  $$T_{vw}=\sum_{s=0}^{S_{vw}-1}F_{vw}(s)-S_{vw}-\frac{\sum_{u\in U}\lambda_{u}Y_{uvw}}{\lambda_{w}}\left(\sum_{s=0}^{|w|S_{vw}-1}F_{w}(s)-|w|S_{vw}\right)$$

Proof The proof for expected SVC inventory level in steady state is similar to that the expected pool inventory level in steady state.

$$I_{vw}=\sum_{s=0}^{S_{vw}-1}(S_{vw}-s)P\{N_{vw}=s\}$$

$$I_{vw}=\sum_{s=0}^{S_{vw}-1}F_{vw}(s)$$

where

$$F_{vw}(s)=\sum_{m=0}^{s}P(N_{vw}=m)$$

By the model assumptions, backorders can only occur if all SVCs in a pool are out of stock. $S_{w}=\sum_{v\in V}S_{vw}=|w|S_{vw}$ is the total base-stock level of the pool w. Then the expected backorder level in steady state at each $SVC_{v}$ in pool w is

$$B_{vw=}\left(\frac{\sum_{u\in U}\lambda_{u}Y_{uvw}}{\lambda_{w}}\right)B_{w}.$$

$$B_{vw}=\left(\frac{\sum_{u\in U}\lambda_{u}Y_{uvw}}{\lambda_{w}}\right)\left(\lambda_{w}L_{w}+\left(\sum_{s=0}^{|w|S_{vw}-1}F_{w}(s)-|w|S_{vw}\right)\right)$$

hence

$$B_{vw}=\sum_{u\in U}\lambda_{u}Y_{uvw}L_{w}+\frac{\sum_{u\in U}\lambda_{u}Y_{uvw}}{\lambda_{w}}\left(\sum_{s=0}^{|w|S_{vw}-1}F_{w}(s)-|w|S_{vw}\right)$$

The behaviour of a Markovian queue in steady state and the consequences of following the (S-1,S) policy help determine the balance equation at a SVC. The balance equation for the SVC is

$$T_{vw}+B_{vw}+S_{vw}-I_{vw}=E[N_{vw}]$$

The expected number of requests filled from on-hand inventory in steady state is given by $S_{vw}-I_{vw}$. The steady state outflow for the SVC is given by $T_{vw}+B_{vw}+S_{vw}-I_{vw}$ and the steady state inflow is given by $E[N_{vw}]$. Therefore, the distribution of the outstanding orders in steady state $N_{vw}$, defined to be the number of items that a SVC has ordered but not yet arrived, is required to determine the inventory, backorder and transshipment levels.

At any given time t, the number of outstanding orders from a SVC comprises of:

(a) plant backorders due to the SVC at time $t-\alpha_{w}$ (this quantity will not reach the SVC before t) and

(b) the number of new orders made during the interval $\left(t-\alpha_{w},t\right)$.

Order arrival at the plant follows a Poisson process and the plant follows First Come First Served (FCFS) rule to the process orders. Hence, plant backorders can be randomly split as in [2]. Consequently, the probability that a backorder emanates from a particular SVC is proportional to the plant demand rate. In steady state, the expected value a SVC backorder is given by $\frac{\sum_{u\in U}\lambda_{u}Y_{uvw}}{\lambda_{0}}B_{0}$.

The expected number of new orders made in a time interval of length $\alpha_{w}$ is $\lambda_{w}\alpha_{w}$. Therefore the expected number of $N_{vw}$ in steady state is given by:

$$E[N_{vw}]=\frac{\sum_{u\in U}\lambda_{u}Y_{uvw}}{\lambda_{0}}\frac{\rho^{S_{0}+1}}{1-\rho }+\sum_{u\in U}\lambda_{u}Y_{uvw}\alpha_{w}=\sum_{u\in U}\lambda_{u}Y_{uvw}L_{w}$$

Hence

$$T_{vw}=\sum_{u\in U}\lambda_{u}Y_{uvw}L_{w}+\sum_{s=0}^{S_{vw}-1}F_{vw}(s)-S_{vw}-\sum_{u\in U}\lambda_{u}Y_{uvw}L_{w}-\frac{\sum_{u\in U}\lambda_{u}Y_{uvw}}{\lambda_{w}}\left(\sum_{s=0}^{|w|S_{vw}-1}F_{w}(s)-|w|S_{vw}\right)$$

Therefore

$$T_{vw}=\frac{\sum_{u\in U}\lambda_{u}Y_{uvw}}{\lambda_{w}}\sum_{s=0}^{|w|S_{vw}-1}[1-F_{w}(s)]-\sum_{s=0}^{S_{vw}-1}[1-F_{vw}(s)]=\sum_{s=0}^{S_{vw}-1}F_{vw}(s)-S_{vw}-\frac{\sum_{u\in U}\lambda_{u}Y_{uvw}}{\lambda_{w}}\left(\sum_{s=0}^{|w|S_{vw}-1}F_{w}(s)-|w|S_{vw}\right)$$

Note

$$\sum_{s=0}^{|w|S_{vw}-1}F_{w}(s)-|w|S_{vw}=-\sum_{s=0}^{|w|S_{vw}-1}[1-F_{w}(s)]$$

 □

It is obvious that before inventory levels can be evaluated, the distribution of the outstanding number of orders $N_{w}$ and $N_{vw}$ at the pool and SVCs, respectively, have to be determined. The multi-echelon technique for repairable item control (METRIC) approximation [1] is used to determine $N_{w}$ and $N_{vw}$. It applies Palm's theorem [36] and approximates the outstanding order distribution using Poisson distribution with corresponding mean. Using METRIC method distributions are constructed for $N_{w}$ and $N_{vw}$ with matching means.

$$P[N_{w}=m]=\frac{e^{\lambda_{w}L_{w}}{\left(\lambda_{w}L_{w}\right)}^{m}}{m!}$$

and

$$F_{w}(s)=\sum_{m=0}^{s}\frac{e^{\lambda_{w}L_{w}}{\left(\lambda_{w}L_{w}\right)}^{m}}{m!}$$

Similarly

$$P[N_{vw}=m]=\frac{e^{-\sum_{u\in U}\lambda_{u}Y_{uvw}L_{w}}{\left(\sum_{u\in U}\lambda_{u}Y_{uvw}L_{w}\right)}^{m}}{m!}$$

and

$$F_{vw}(s)=\sum_{m=0}^{s}\frac{e^{-\sum_{u\in U}\lambda_{u}Y_{uvw}L_{w}}{\left(\sum_{u\in U}\lambda_{u}Y_{uvw}L_{w}\right)}^{m}}{m!}$$

In the above, $\sum_{u\in U}\lambda_{u}Y_{uvw}$ represents demand assignment to SVC v in pool w. $L_{w}$ represents expected lead time for replenishment, this is made up of plant delay and the deterministic plant to SVC lead time. Note that $L_{w}$ here is the same as that for the pool; this is because it is assumed that lateral transshipment between SVCs in a pool is instantaneous. Thus, the lateral transshipment times are negligible.

METRIC discards the dependence between successive replenishment times from Plant to SVC. These replenishment times depend on the plant inventory, hence they are not independent. [24] showed that in general METRIC works for systems that have low SVC demand compared to overall demand. The METRIC approximation performs well in such instances mainly due to the fact that successive replenishment times to a SVC is reduced as a result of many other order arrivals at the plant from other SVCs. [8] established that METRIC is a very good approximation for our system. This is because the demand occurring at each SVC is low compared to total demand.

The expected levels for on-hand inventory, LT and backorder in steady state lead to the following reformulation of the problem.

(15) $$\min\sum_{w\in W}\sum_{v\in V}\{f_{vw}X_{vw}+(h_{vw}+q_{vw})\sum_{s=0}^{S_{vw}-1}F_{vw}(s)-q_{vw}S_{vw}+\sum_{u\in U}\left(\left(p_{vw}L_{w}+d_{uvw}\right)\lambda_{u}Y_{uvw}\right)+\left(p_{vw}-q_{vw})\frac{\sum_{u\in U}\lambda_{u}Y_{uvw}}{\lambda_{w}}\left(\sum_{s=0}^{|w|S_{vw}-1}F_{w}(s)-|w|S_{vw}\right)\right\}+h_{0}[S_{0}-\frac{\rho }{1-\rho }(1-\rho^{S_{0}})]$$

Subject to

(16) $$\sum_{v\in V}Y_{uvw}=1,\text{ for any }u\in U$$

(17) $$Y_{uvw}\leq a_{uvw}X_{vw},\text{ for any }u\in U,v\in V\;S_{vw}\leq C_{vw},\text{ for any }v\in V\;S_{w}\leq C_{w}=|w|C_{vw},\text{ for any }w\in W\;S_{0}\leq C_{0}\left[L_{w}-\tau \right]\leq \frac{\sum_{s=0}^{|w|S_{vw}-1}[1-F_{w}(s)]}{\lambda_{w}}\;S_{vw},S_{w},S_{0}\geq 0\text{ integer, for any }v\in V\;X_{vw}\in \{0,1\}\text{ for any }v\in V\;Y_{uvw}\in \{0,1\}\text{ for any }u\in U$$

## 4 Model properties

We exploit the model structure to determine mathematical properties of the model in this section

### 4.1 Upper bound for plant basestock level

The following model characteristic ensures that the plant's base-stock level has an upper bound in many instances, it describes the model when backorder cost is set to zero and the requirement on response time exceeds the deterministic lead time between pool and plant. This characteristic follows from the model by [9]. Our model reduces to that of [9] if each pool is considered as a SVC.

Proposition 4.1.1 *Given that the objective function* (15) *is strictly monotone increasing in* $S_{0}$ *. If* $p_{vw}=0$ *,* $L_{w_{max}}=W_{0}+\max_{w\in W}\{\alpha_{w}\}$ *, and* $L_{w_{max}}\leq \tau$ *, then there exists an upper bound to the plant's base-stock level* $S_{0}$ *, which is represented by:*

$$S_{0}^{max}=\min\{S_{0}\geq 0:L_{w_{max}}\leq \tau \}$$

Proof The response time constraint (17) can also be written as can also be written as

$$\left[L_{w}-\tau \right]\leq \frac{\sum_{s=0}^{|w|S_{vw}-1}[1-F_{w}(s)]}{\lambda_{w}}.$$

The requirement $L_{w_{max}}\leq \tau$ infers that for any value of $S_{vw}$, LHS of (17) will be nonpositive. By definition, $0\leq F_{w}(s)\leq 1$ and $1-F_{w}(s)\geq 0$, therefore at $S_{0}^{max}$, $\frac{\sum_{s=0}^{|w|S_{vw}-1}[1-F_{w}(s)]}{\lambda_{w}}\geq 0$ always hold, implying that the service constraint is always satisfied at $S_{0}^{max}$. Thus the only constraints on $S_{0}$ are the service constraint and requirement of nonnegativity, hence the feasibility of $S_{0}^{max}$ holds with respect to other decision variables. A consequence of the objective function (15) being strictly monotone increasing in $S_{0}$, is that suboptimal solutions will be obtained for all instances where $S_{0}>S_{0}^{max}$. □

The proposition above states that if backorder costs are nonexistent and the requirement on response time is greater than all deterministic plant to pool lead times, then exists a plant base-stock level which guarantees that all service constraints will be satisfied with all SVCs holding zero stock. The optimal base-stock level of the plant will not surpass this level.

This model characteristic, the presence capacity constraints and our assumption of low demand across the entire system, imply that we only need to consider stock levels within a small interval in order to ensure the desired service level. The work by [19] made use of similar characteristics.

For this model, the capacity constraint on plant implies that we can fix the base-stock level of the plant to each feasible value and then solve some continuous problems. The continuous solution with the best cost becomes the original problem's optimal solution. With plant base-stock level fixed, all terms depending only on plant base-stock $S_{0}$ become constants.

### 4.2 Lagrange relaxed model

We relax assignment constraints (16) the service constraints (17). Using $\pi_{u}$ and $\theta_{vw}$ to denote the corresponding dual multipliers for constraints (16) and (17) respectively, the following Lagrangian Dual problem is obtained:

$$\max_{\theta \geq 0,\pi }\min_{X,Y,S}\sum_{w\in W}\sum_{v\in V}\{f_{vw}X_{vw}+(h_{vw}+q_{vw})\sum_{s=0}^{S_{vw}-1}F_{vw}(s)-q_{vw}S_{vw}+(p_{vw}-q_{vw}+\theta_{vw})\frac{\sum_{u\in U}\lambda_{u}Y_{uvw}}{\lambda_{w}}\sum_{s=0}^{|w|S_{vw}-1}F_{w}(s)-\frac{\sum_{u\in U}\lambda_{u}Y_{uvw}}{\lambda_{w}}(p_{vw}-q_{vw}+\theta_{vw})|w|S_{vw}+\sum_{u\in U}\left(((p_{vw}+\theta_{vw})L_{w}+d_{uvw}+\theta_{vw}\alpha_{w}-\theta_{vw}\tau )\lambda_{u}-\pi_{u}\right)Y_{uvw}\}+\sum_{u\in U}\pi_{u}$$

Subject to

$$Y_{uvw}\leq a_{uvw}X_{vw},\text{ for any }u\in U,v\in V\;S_{vw}\leq C_{vw}X_{vw},\;\text{for any}\;v\in V,w\in W\;S_{0}\leq C_{0}\;X_{vw}\in \{0,1\},\;\text{for any}\;v\in V\;Y_{uvw}\in \{0,1\},\text{ for any }u\in U\;S_{vw},S_{w},S_{0}\geq 0\text{ integer , for any }v\in V,w\in W$$

Fixing all the $X_{vw}$ ∀ v∈V further reduces the problem to

$$\max_{\theta \geq 0,\pi }\min_{Y,S}\sum_{w\in W}\sum_{v\in V}\{f_{vw}+(h_{vw}+q_{vw})\sum_{s=0}^{S_{vw}-1}F_{vw}(s)-q_{vw}S_{vw}+(p_{vw}-q_{vw}+\theta_{vw})\frac{\sum_{u\in U}\lambda_{u}Y_{uvw}}{\lambda_{w}}\sum_{s=0}^{|w|S_{vw}-1}F_{w}(s)-\frac{\sum_{u\in U}\lambda_{u}Y_{uvw}}{\lambda_{w}}(p_{vw}-q_{vw}+\theta_{vw})|w|S_{vw}+\sum_{u\in U}\left(((p_{vw}+\theta_{vw})L_{w}+d_{uvw}+\theta_{vw}\alpha_{w}-\theta_{vw}\tau )\lambda_{u}-\pi_{u}\right)Y_{uvw}\}+\sum_{u\in U}\pi_{u}$$

Subject to

$$Y_{uvw}\leq a_{uvw},\text{for any,}u\in U\;0\leq S_{vw}\leq C_{vw}\;\text{for any,}v\in V\;Y_{uvw}\in \{0,1\}\;\text{for any,}u\in U\;S_{vw}\;\text{integer}\;\text{for any,}v\in V$$

Note that $p_{vw}-q_{vw}>0$, that is the backorder cost is always higher that the LT cost. If this were not so, there wouldn't be any reason to prefer LT to backorder.

For given values of Lagrange multipliers, the problem is decomposed by candidate SVC locations and associated pools into subproblems with the following form (for each v∈V, w∈W):

$$\min_{Y,S}(h_{vw}+q_{vw})\sum_{s=0}^{S_{vw}-1}F_{vw}(s)-q_{vw}S_{vw}+(p_{vw}-q_{vw}+\theta_{vw})\frac{\sum_{u\in U}\lambda_{u}Y_{uvw}}{\lambda_{w}}\sum_{s=0}^{|w|S_{vw}-1}F_{w}(s)-\frac{\sum_{u\in U}\lambda_{u}Y_{uvw}}{\lambda_{w}}(p_{vw}-q_{vw}+\theta_{vw})|w|S_{vw}+\sum_{u\in U}\left(((p_{vw}+\theta_{vw})L_{w}+d_{uvw}+\theta_{vw}\alpha_{w}-\theta_{vw}\tau )\lambda_{u}-\pi_{u}\right)Y_{uvw}$$

Subject to

$$Y_{uvw}\leq a_{uvw},\text{for any }u\in U\;0\leq S_{vw}\leq C_{vw}\text{ for any }v\in V\;Y_{uvw}\in \{0,1\}\text{ for any }u\in U\;S_{vw}\text{ integer, for any }v\in V$$

The solution of the above subproblem depends on the limited storage capacities at the SVCs. This suggests that the possible values of $S_{vw}$ in the optimal solution lie between 0 and $C_{vw}$ and also the possible values of $S_{w}$ in the optimal solution lie between O and $C_{w}$. In real life situations, the feasible interval is small even without storage limits since the management may have limited budget for inventory in storage as a result of high costs. Thus a likely approach to solving the subproblem is to fix $S_{vw}$ and $S_{w}$ to each feasible value. Furthermore, the assignment variable $Y_{uvw}$ is relaxed and allowed to be continuous in the interval [0,1]. This continuous relaxation results to a lower bound when we adopt the approach of Lagrangian relaxation.

Fixing the values of $S_{vw}$ and $S_{w}$ reduces the continuous subproblem to a nonlinear problem of the form:

(18) $$\min g(\sum_{u\in U}\lambda_{u}Y_{uvw})+\sum_{u\in U}r_{uvw}Y_{uvw}$$

Subject to

$$0\leq Y_{uvw}\leq 1$$

where

$$g\left(\sum_{u\in U}\lambda_{u}Y_{uvw}\right)=(h_{vw}+q_{vw})\sum_{s=0}^{S_{vw}-1}F_{vw}\left(s,\sum_{u\in U}\lambda_{u}Y_{uvw}\right)+(p_{vw}-q_{vw}+\theta_{vw})\frac{\sum_{u\in U}\lambda_{u}Y_{uvw}}{\lambda_{w}}\sum_{s=0}^{|w|S_{vw}-1}F_{w}(s,\lambda_{w})$$

$$F_{vw}(s,\sum_{u\in U}\lambda_{u}Y_{uvw})=\sum_{m=0}^{s}\frac{e^{-\sum_{u\in U}\lambda_{u}Y_{uvw}L_{vw}}{\left(\sum_{u\in U}\lambda_{u}Y_{uvw}L_{vw}\right)}^{m}}{m!}\;F_{w}(s,\lambda_{w})=\sum_{m=0}^{s}\frac{e^{-\lambda_{w}L_{vw}}{\left(\lambda_{w}L_{vw}\right)}^{m}}{m!}$$

and

$$r_{uvw}=((p_{vw}+\theta_{vw})L_{w}+d_{uvw}-\theta_{vw}\tau )\lambda_{u}-\pi_{u}-\frac{\lambda_{u}}{\lambda_{w}}(p_{vw}-q_{vw}+\theta_{vw})|w|S_{vw}$$

Let $\sum_{u\in U}\lambda_{u}Y_{uvw}=a$, obtaining solutions for the subproblem is dependent on the characteristics of g(a) to be discussed below.

We begin by establishing convexity of the subproblem.

Proposition 4.2.1 g(a) *is convex in a when* a≥0 *.*

Proof $\sum_{u\in U}\lambda_{u}Y_{uvw}=a$ is demand assignment to the SVC with base-stock level $S_{vw}$, and $\lambda_{w}$ is the total demand at pool w.

$$g(a)=(h_{vw}+q_{vw})\sum_{s=0}^{S_{vw}-1}F_{vw}(s)+(p_{vw}-q_{vw}+\theta_{vw})\frac{a}{\lambda_{w}}\sum_{s=0}^{S_{w}-1}F_{w}(s)$$

Taking the derivatives of g(a) with respect to a

$$\frac{d}{da}g(a)=(h_{vw}+q_{vw})\sum_{s=0}^{S_{vw}-1}\frac{d}{da}F_{vw}(s)+(p_{vw}-q_{vw}+\theta_{vw})\frac{\sum_{s=0}^{|w|S_{vw}-1}F_{w}(s)}{\lambda_{w}}$$

where

$$F_{vw}(s)=\sum_{m=0}^{s}\frac{e^{-aL_{w}}{\left(aL_{w}\right)}^{m}}{m!}$$

$$\frac{d}{da}g(a)=(h_{vw}+q_{vw})\sum_{s=0}^{S_{vw}-1}\sum_{m=0}^{s}\frac{1}{m!}(-L_{w}e^{-aL_{vw}}{\left(aL_{w}\right)}^{m}+e^{-aL_{w}}m{\left(a\right)}^{m-1}L_{w}^{m})+(p_{vw}-q_{vw}+\theta_{vw})\frac{\sum_{s=0}^{|w|S_{vw}-1}F_{w}(s)}{\lambda_{w}}$$

$$\frac{d}{da}g(a)=-(h_{vw}+q_{vw})\sum_{s=0}^{S_{vw}-1}\frac{L_{w}e^{-aL_{vw}}{\left(aL_{w}\right)}^{s}}{s!}+(p_{vw}-q_{vw}+\theta_{vw})\frac{\sum_{s=0}^{|w|S_{vw}-1}F_{w}(s)}{\lambda_{w}}$$

$$\frac{d^{2}}{da^{2}}g(a)=(h_{vw}+q_{vw})\frac{L_{w}^{2}{\left(aL_{w}\right)}^{S_{vw}-1}e^{-aL_{w}}}{(S_{vw}-1)!}\geq 0$$

This establishes the convexity of g(a) with respect to demand assignment. □

Proposition 4.2.2 *The continuous subproblem* (18) *is a convex.*

Proof This follows from the proposition above and the fact that a sum of convex functions is also convex. □

Proposition 4.2.3 *Our model is convex.*

Proof We showed convexity of the dual problem for fixed multiplier values and continuous assignment variables. Thus, the dual objective is convex when $\theta_{vw},\pi_{u}=0$ and $0\leq Y_{uvw}\leq 1$. Also the dual objective is equal to the primal objective when $\theta_{vw},\pi_{u}=0$ and $0\leq Y_{uvw}\leq 1$. A nonlinear mixed integer problem is convex if it's corresponding integer relaxed problem is convex, hence the objective function of our model is convex. The constraints on response time and the assignment constraints are the complicating constraints associated with our model.

The response time constraint in our model depends only on the variable $S_{w}=|w|S_{vw}$ and can be written as

$$L_{w}-\tau +\frac{1}{\lambda_{w}}\sum_{s=0}^{S_{w}-1}\left(F_{w}(s)-1\right)\leq 0$$

Let

$$\overset{¯}{J}(S_{w})=L_{w}-\tau +\frac{1}{\lambda_{w}}\sum_{s=0}^{S_{w}-1}\left(F_{w}(s)-1\right)$$

$$△\overset{¯}{J}(S_{w})=\overset{¯}{J}(S_{w}+1)-\overset{¯}{J}(S_{w})=\frac{1}{\lambda_{w}}(F_{w}(S_{w})-1)=-\frac{1}{\lambda_{w}}(1-F_{w}(S_{w}-1))<0$$

where $△\overset{¯}{J}(S_{w})$ is the first difference of $\overset{¯}{J}(S_{w})$.

$${△}^{2}\overset{¯}{J}(S_{w})=△\overset{¯}{J}(S_{w}+1)-△\overset{¯}{J}(S_{w})=\frac{1}{\lambda_{w}}(F_{w}(S_{w}+1)-F_{w}(S_{w}))=\frac{1}{\lambda_{w}}\left(\sum_{m}^{S_{w}+1}P[N_{w}=m]-\sum_{m}^{S_{w}}P[N_{w}=m]\right)=\frac{1}{\lambda_{w}}\left(P[N_{w}=S_{w}+1]\right)>0$$

where ${△}^{2}\overset{¯}{J}(S_{w})$ is the second difference of $\overset{¯}{J}(S_{w})$. Thus the inequality constraint of our model is convex.

The equality constraint can be written as $1-\sum_{v\in V}Y_{uvw}=0$. $1-\sum_{v\in V}Y_{uvw}$ is the sum of a linear function and a constant, thus it is affine.

The objective function and the constraints on response time are convex, while the equality constraint is affine. This establishes the convexity of our model. □

Convexity implies solutions to continuous subproblems can be obtained by means of standard optimization solvers. The solution to our model can be obtained using GAMS software, thus there is no urgency to immediately develop any specialized heuristics for solving it. However, future studies will consider the development of specialised heuristics and compare the solution obtained with solutions obtained using existing software.

## 5 Computational experiments and discussions

In this section computation experiments are designed and implemented to examine our model's properties. We implement our model using GAMS 32.1.0 and the solver used is DICOPT, DICOPT is used for solving MINLP models. Three data sets comprising of 37 nodes, 109 nodes and 181 nodes are used. The 37 node data set represents the 36 state capitals and Federal Capital Territory (FCT) in Nigeria. The 109 node data set contains the FCT and the 3 most populous cities in each of the 36 states. The 181 node data set contains the FCT and the 5 most populous states in each state. Each node is considered as a potential SVC location and as a demand node. The plant is located at the FCT for all data sets used, other nodes are considered as candidate SVC locations. Our pools are defined by the six geopolitical zones in Nigeria, thus SVCs located in the same geopolitical zone are pooled together. The population data is gotten from the 2006 census [38]. Nodes in same geopolitical zone form a pool. This problem is similar to real-world problem of a manufacturer with the objective of setting up a plant and service centers in a region where customers are dispersed geographically. The major novelty of this paper is the incorporation of LT into a two-echelon inventory-location system with RTR. It then follows that the comparison of our model with a model without LT is our major result in this section. The experiments of interest in this section are: examination of how our model fares when compared with a model without LT, effect of capacity constraints, effect of base-stock levels, and effect of response times. We believe these experiments are sufficient to draw significant managerial insights for our model.

### 5.1 Parameter setting

In this subsection, we present the various parameter settings used in our computational experiments. Following same ideas behind the 150-node data set of [39], the fixed cost of siting a SVC at location v in pool w, $f_{vw}$, is set to 10000 for all locations. The per unit cost of inventory storage at the plant per unit time and at any SVC per unit time, $h_{0}$ and $h_{vw}$ respectively, are set to 50. The lateral transshipment cost per unit inventory at a SVC, $q_{vw}$, is set to 25, and the per unit cost of backorder at a SVC per unit time, $p_{vw}$, is set to 70. This is similar to what is obtainable in practice, the backorder cost is greater that the holding and lateral transshipment costs in order to make backorders the least appealing means of fulfilling customer demand. Also, in practice, the lateral transshipment cost is a fraction of the holding cost in systems that have slow moving items and negligible lateral transshipment times. Although, the lateral transshipment cost is less than the holding cost, the response time requirements of customers constrain supply chain managers to ensure that they keep a certain level of inventory on hand. The transportation cost from a SVC at location v in pool w to customer u, $d_{uvw}$, is derived by multiplying the distance between SVCs and customers by $10^{-1}$. The demand rate of customer u, $\lambda_{u}=$, is obtained by multiplying the population at node u by $10^{-5}$, the population data is obtained from the 2006 Nigerian census. Demand is constrained to be no greater than 10 for nodes with very large population. This follows because this study is applicable to slow moving items. The plant's utilisation rate, *ρ*, is set to 0.5 and 0.9 respectively. The average requirement on response time, *τ*, is varied between 0.2 and 0.6. To obtain the deterministic lead time for transportation from the plant to a SVC, the distance from plant to the SVC is divided by 2400. The plant to pool w deterministic lead time, $\alpha_{w}$, is set to the maximum of all plant to SVC lead times for all SVCs in pool w. The maximum distance permitted between a customer and the SVC assigned, $d_{max}$, is set to 100 km and 150 km. We vary $C_{vw}$ between 1 and 10 in order to closely mirror what is obtainable in practice for items that are expensive and slow moving.

### 5.2 Model performance

Here we are interested in how our model fares when compared with the model of [9], we describe their model as the model without LT. A major contribution of this work is the incorporation of LT into the model considered by [9], thus, it makes sense to compare our model with their model under various scenarios. This test is conducted using the 37 nodes, 109 nodes and 181 nodes data sets for $\left(\rho =UR=(0.9,0.5),\tau =RTR=(0.5,0.3,0.2),d_{max}=(150,100\right)$. We take note of the value of our model's objective function (OBJ) and the value of the objective function of the model without LT (OBJWLT). We also note the number of SVCs opened for our model (SVCs) and the number of SVCs opened for the model without LT (SVCsWLT).

The results are summarised in Table 1. For the 37 node data set, the entire system cost for our LT model is lower compared to the model without LT when $d_{max}=150$, while the model without LT performs better than our model when $d_{max}=100$. Generally, our model gives lower costs for as long as $d_{max}\geq 120$. Also in all instances tested for the 109 nodes data set and the 181 nodes data set respectively, the total system cost of our LT model was lower than that of the model without LT. This shows that our model is suitable for systems having many nodes. Also in most instances fewer SVCs are required for our model compared to the model without LT. Thus the incorporation of LT results to lower system cost in general. This happens even when our model has more SVCs opened. Also, the total cost increases as $d_{max}$ reduces from 150 to 100, this is because more facilities need to be opened as the coverage distance reduces.

**Table 1:** Model performance.

S/N	NODES	UR	RTR	dmax	OBJ	OBJWLT	SVCs	SVCsWLT
1	37	0.9	0.5	150	150150.766	232026.849	19	18
2	37	0.5	0.5	150	150306.274	231638.484	19	18
3	37	0.9	0.3	150	150150.766	232026.849	19	18
4	37	0.5	0.3	150	150306.274	231802.101	19	18
5	37	0.9	0.2	150	150150.766	232026.849	19	18
6	37	0.5	0.2	150	150306.274	232011.199	19	18
7	37	0.9	0.5	100	236693.918	277923.761	28	26
8	37	0.5	0.5	100	236848.895	277516.981	28	26
9	37	0.9	0.3	100	236693.918	277887.272	28	26
10	37	0.5	0.3	100	236848.895	277516.981	28	26
11	37	0.9	0.2	100	236693.918	277923.761	28	26
12	37	0.5	0.2	100	236848.895	277516.981	28	26
13	109	0.9	0.5	150	118780.906	369742.764	25	23
14	109	0.5	0.5	150	118881.992	370897.5926	25	23
16	109	0.5	0.3	150	118923.379	371742.966	25	23
17	109	0.9	0.2	150	118748.428	370498.053	25	23
19	109	0.9	0.5	100	237908.64	424427.681	37	32
20	109	0.5	0.5	100	238086.585	424997.982	37	32
21	109	0.9	0.3	100	237913.208	424883.921	37	32
22	109	0.5	0.3	100	238068.574	424492.061	37	32
23	109	0.9	0.2	100	237931.935	424492.061	37	32
24	109	0.5	0.2	100	238072.758	424523.052	37	32
25	181	0.9	0.5	150	101624.661	444404.099	23	24
26	181	0.5	0.5	150	101668.753	443361.185	23	23
27	181	0.9	0.3	150	101626.211	443491.512	23	23
28	181	0.5	0.3	150	101783.097	443361.185	23	23
29	181	0.9	0.2	150	101597.273	443185.051	23	23
30	181	0.5	0.2	150	101648.105	443611.699	23	23
31	181	0.9	0.5	100	309766.416	515944.434	44	34
32	181	0.5	0.5	100	309885.986	515081.961	44	35
33	181	0.9	0.3	100	309706.022	514487.72	44	36
34	181	0.5	0.3	100	309864.146	517557.221	44	34
35	181	0.9	0.2	100	309724.372	515508.839	44	34
36	181	0.5	0.2	100	309902.615	514899.816	44	34

### 5.3 Effect capacity on minimum cost

**Figure 2:**
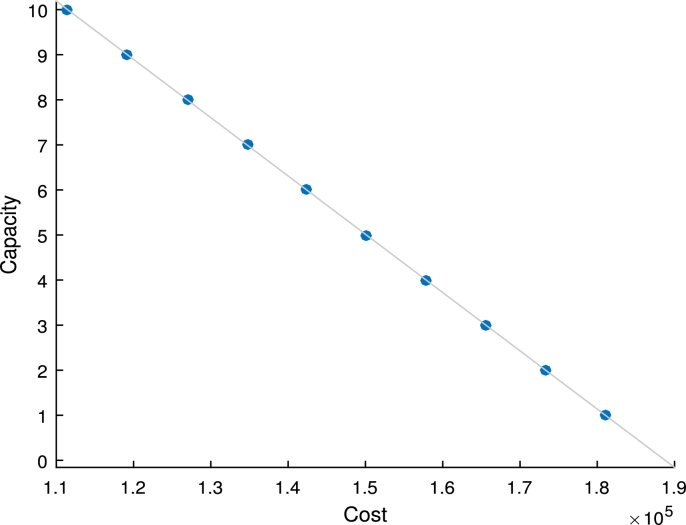
Effect of SVC capacity on cost.

We utilise the 37 node data set for this experiment. The maximum distance from a customer to a SVC is set to 150 km, the utility rate is set to 0.9 and the response time is set to 0.2. The capacity at each facility is varied from 1 to 10. This is because capacity constraints can also be viewed as budget constraints and it's not always optimal to stock many slow moving expensive items. The minimum cost is taken in each instance. The minimum costs are plotted against the various capacity levels. The resulting graph is depicted by Fig. 2 which shows that increase in capacity results to a significant decrease in total system cost. This is because an increase in capacity results to higher stock levels which consequently leads to a decrease in lateral transshipment costs and backorder costs. Since the range of capacity is usually small for the problem considered and base-stock level is constrained by capacity, base-stock levels can be fixed to any of it's feasible values until a value which satisfies the objective of the decision maker is attained.

### 5.4 Effect of stock level on minimum response time

**Figure 3:**
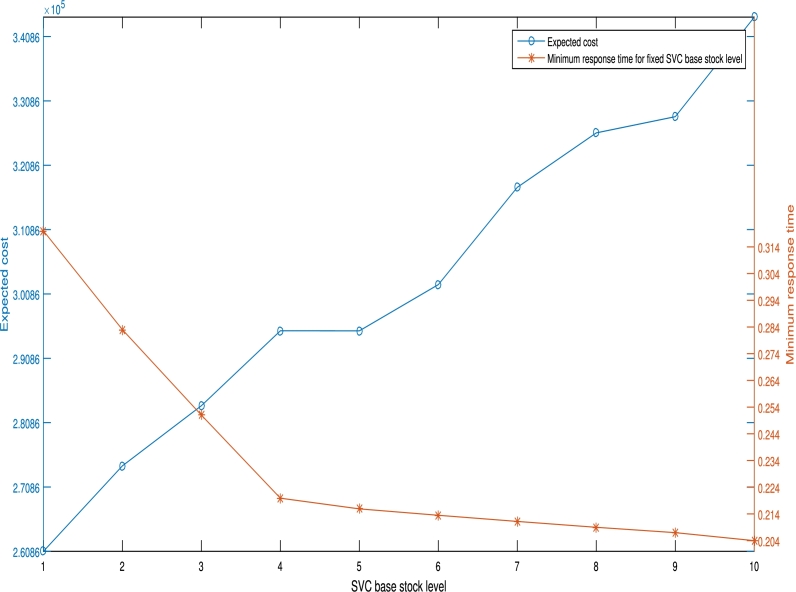
Effect of SVC stock level on minimum response time.

**Figure 4:**
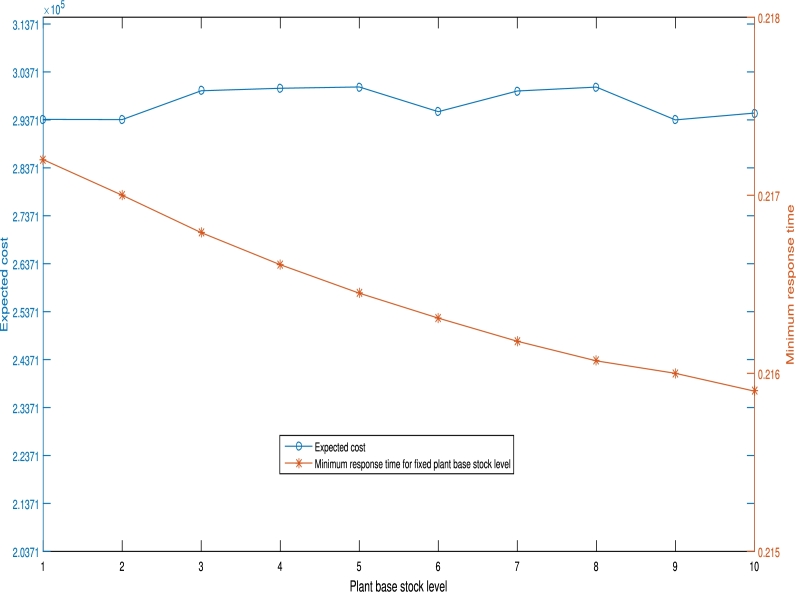
Effect of plant stock level on minimum response time.

We utilize the 37 node data set for this experiment. The stock levels at SVCs and at the plant are varied from 1 to 10. For each feasible point the stock levels are fixed at the SVCs and at the plant. In each instance, the minimum feasible response time and the corresponding minimum cost are taken. The minimum response time and minimum costs are plotted against the stock level for plant and SVC respectively. The resulting graphs are shown in Fig. 3 and Fig. 4 respectively. From Fig. 3, the minimum feasible response times reduce as the SVC stock level increases from 1 to 10 while the corresponding minimum costs increase. The minimum service time reduces because an increase in stock level results to reduced waiting time. The increase in cost occurs because increasing stocking levels across all SVCs imply an increase in inventory holding cost across all facilities. Thus the decision maker can decide on the SVC stock level that gives the desirable response time subject to budget constraints. From Fig. 4, the minimum response time reduces as the plant's base-stock level increases while the corresponding costs remain consistent with little or no change. The implication of this is that holding more plant results to a system wide reduction in feasible response time values with little or no increase in total system cost. The increase in plant holding costs as a result of a few extra units is usually negligible when compared to the total system costs. The managerial insight drawn from this is that increasing the plant's stock level is preferable if the objective is to find a balance between minimising response times and minimising costs.

### 5.5 Effect of response time

**Figure 5:**
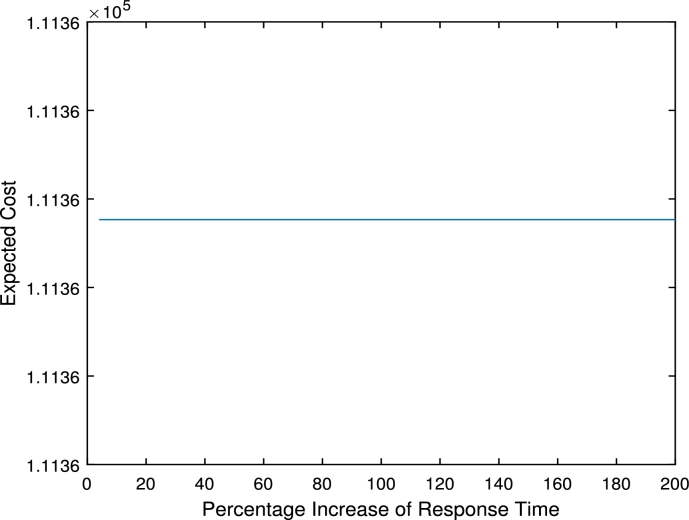
Effect of response time.

This experiment considers the behaviour of the model with LT as response time varies. The 37-node dataset is used with (*ρ*) set to 0.9, $d_{max}$ set to 150 kilometers. The requirement on response time is varied between 0.2 and 0.6 time units by gradually increasing 0.2 by 2%,4%,...,200% and for each value, the total cost expected is also recorded. Then we plotted the total costs expected against the percentage increase. From Fig. 5 it is observed that the graph for response time is consistent, i.e. there is little or no change in cost when the requirement on response time is varied among its feasible points. The consistency of expected cost with varying response times is a consequence of lateral transshipment which ensures that the response time constraint is uniform for all service centers in a pool. This implies that for our model, the response time can be tightened or slackened within the feasible bound and have little or no effect on the expected cost. For the model without LT, [9] showed that tightening of response times resulted to increase in expected costs.

## 6 Conclusion

This study incorporates lateral transshipment into a single item two echelon spare parts supply chain with service consideration. Lateral transshipment can only occur between service centers in same pool. The entities in our model consist of a set of possible service center locations, a set of pools and a collection of customers. This study considered the following costs: fixed costs of opening service centers at candidate locations, inventory costs emanating from holding stock at the service centers and at the plant, lateral transshipment costs which occur when stock is moved between facilities on same echelon, the costs associated with backordering, and shipping costs which arise when items are sent from service centers to their assigned customers. For the system considered, we derive expected inventory levels in steady state, expected lateral transshipment levels in steady state, and expected backorder levels in steady state. The formulated model is a mixed integer nonlinear programming problem and can be regarded as an extension of a two-echelon location-inventory model with service constraints and a requirement on response time.

Using Lagrange relaxation, the model was decomposed and convexity of our model was established. Convexity of our model implies that we can obtain solutions to the model using optimization solvers. The model was solved with GAMS 32.1.0 using datasets with 37, 109, and 181 nodes. In most instances our model returned lower costs when compared with a model without lateral transshipment. Fewer service centers were required for our model when compared with a model without a model without lateral transshipment. We established that an increase in capacity leads to a decrease in total system costs, this however, is also subject to the budget available. In addition, we showed that increase in service center stock level resulted to increase in expected cost and reduction in minimum response time, while increase in plant stock level resulted to reduction in minimum response time with consistency in expected cost. Thus, the decision maker can decide on the stock level that gives the desirable response time subject to budget constraints. Finally, we showed that a major effect of using lateral transshipment is that the expected cost remains consistent when the response time is varied between its feasible points.

This work can be extended in several directions. The problem considered in this study was solved using GAMS. A disadvantage of using commercial solvers is that they may not perform well when solving large problems. Although this limitation was not evident with the use of GAMS for this study, it may become evident with different variants of the problem considered. Also there may be other solution methods which will perform better than GAMS. Thus, the development of heuristic solutions for the problem would be an interesting direction for future research. The consideration of the problem for systems when lateral transshipment times are non-negligible is also another possible future research direction. This is because the assumption of negligible transshipment times made in this research may not be applicable in industries such as dredging, where some service parts are too heavy for transportation by air and the lateral transshipment times are not negligible compared to lead times. In addition, relaxation of the assumption of (S-1,S) inventory policy would be an exciting extension of this work. This relaxation will allow batch ordering and would increase the applicability of this model to more supply chain designs.

## Declarations

### Author contribution statement

Unanaowo Nyong Basseya: Conceived and designed the experiments; Contributed materials, analysis tools or data. Samuel Chiabom Zelibe: Performed the experiments; Analyzed and interpreted the data; Wrote the paper.

### Funding statement

This research did not receive any specific grant from funding agencies in the public, commercial, or not-for-profit sectors.

### Data availability statement

Data included in article/supplementary material/referenced in article.

### Declaration of interests statement

The authors declare no conflict of interest.

### Additional information

No additional information is available for this paper.
